# Operating with Data - Statistics for the Cardiovascular Surgeon: Part II. Association and Risk

**DOI:** 10.21470/1678-9741-2018-0247

**Published:** 2018

**Authors:** Gabriel Romero Liguori, Luiz Felipe Pinho Moreira

**Affiliations:** 1 Laboratório de Cirurgia Cardiovascular e Fisiopatologia da Circulação (LIM-11), Instituto do Coração (InCor), Hospital das Clínicas HCFMUSP, Faculdade de Medicina, Universidade de São Paulo, São Paulo, SP, Brazil.

In the last issue of the Brazilian Journal of Cardiovascular Surgery (BJCVS) we published the first editorial of this editorial series entitled "Operating with Data - Statistics for the Cardiovascular Surgeon". There, we addressed the fundamental concepts required for understanding biostatistics^[1]^. Now, we will discuss association and risk, two interconnected and fundamental entities within biostatistics. Again, we will not focus on formulas or in the mathematical theory, we will rather try to explain, in an easy and straightforward manner, the most relevant concepts and how they can be applied, making use of with practical examples.

## What is an Association?

Although the word association may represent several different ways in which two things can be connected, sometimes even being interchangeably used with the term correlation, herein we define association as the way two qualitative variables are related to each other. Another way one can see association is as being a comparison between the proportions of two or more groups (each qualitative variable may present several groups). Indeed, this definition is not wrong, but we will opt to use the term comparison, as well as correlation, for other kinds of relationships between variables, which will be described in the future editorials.

To make the concept clearer, let's make use of a practical example. In a previous issue of the BJCVS, Dayan et al. analyzed the outcomes of coronary artery bypass graft (CABG) with and without aortic cross-clamp (AXC)^[2]^. One of the dependent variables analyzed by the authors was the need for postoperative prolonged ventilatory support (PVS). Among the 1145 patients undergoing CABG, 988 were submitted to AXC and 157 were not. For those submitted to AXC, 489 required PVS, while this number was 43 for the group without AXC.

One way to represent these findings is simply presenting them as percentages. In the group submitted to AXC, 489/988 *i.e.* 49.5% required PVS, while in the group without AXC only 43/157 *i.e.* 27.4% needed it. However, another way to represent these findings is to use a contingency table, also known as a cross-tabulation or crosstab. A contingency table represents one variable as the rows (usually the independent variable) and the other variable as the columns (usually the dependent variable). In our example, the independent variable is the surgical treatment and the dependent variable is the outcome *i.e.* PVS (Table 1). An important observation is that not always the rows and columns will represent independent and dependent variables since other types of associations, for instance between two diagnostic methods, can be analyzed and one variable is not interfering in the other.

**Table 1:** Contingency table (cross tabulation or crosstab).

		Dependent variable		Total
		PVS	No PVS	
Independent variable	CABG with AXC	489	499	988
	CABG without AXC	43	114	157
**Total**		532	613	1145

Contingency tables can be created with variables containing many groups, not only two as in the example. In case you submitted the patients to three different surgical procedures or in case the intervention can generate three different outcomes (*e.g.* alive without sequelae, alive with sequelae, and dead), it would be necessary to use, respectively, a 3X2 and a 2X3 table. Although most tests can statistically analyze tables despite of their sizes, some essential measures of risk can only be calculated for 2X2 tables, also known as fourfold tables. In this regard, a contingency table is not merely a way to represent the data; it is also a tool to calculate a series of statistical tests and measurements of clinical interest.

## Tests of Association

As for most other kinds of relationship between two sets of data, or two variables, the choice of the statistical test to be used for associations will depend on two main factors: the size and distribution of the sample and the pairing of the data.

## Chi-Squared Test (χ2)

The chi-squared test (χ2) of association is a statistical test that compares the observed frequency (O) to the expected frequency (E) if the proportions for each variable remained the same independently of the other variable. The expected frequency is calculated by multiplying the total frequency of the row and column of a determined cell of the table and dividing this value by the total number of subjects in the study. Taking into consideration Table 1, which represents the actual frequency of observations in our example, the expected frequencies are represented in Table 2.

**Table 2:** Expected frequencies.

		Dependent variable		Total
		PVS	No PVS	
Independent variable	CABG with AXC	(532×988)÷1145 = 459	(613×988)÷1145 = 529	988
	CABG without AXC	(532×157)÷1145 = 73	(613×157)÷1145 = 84	157
**Total**		532	613	1145

By comparing Table 1 (the observed frequencies) and Table 2 (the expected frequencies), the χ2 test of association will give a p-value which is based on the degrees of freedom of the data, determined by the numbers of rows and columns. The details regarding the way this calculation is performed will not be covered in this editorial, but the test can be automatically performed by virtually any statistical package and even free online tools^[3]^. In our example, the *P*-value of the χ2 test is <0.0001, representing a statistically significant association between the independent and the dependent variables i.e. the independent variable does affect the dependent variable. The χ2 test of association indicate if there are unexpected differences, thus association, considering the whole table; it does not, however, indicate where these differences are located and the statistical significance for each of them. To determine the cells which are presenting lower or higher values than expected, as well as the strength of these differences, it is necessary to calculate the residuals, which are standardized and adjusted values following the normal distribution. The calculation of residuals is also not the scope of this editorial, but many statistical software include it together with the χ2 test of association. Herein, to better fit clinical purposes, we will focus on the measures of risk derived from the relationship between the two variables, instead of taking into consideration each isolated cell of the contingency table.

The χ2 test of association is an easy and practical statistical test to be used when samples are large, present a normal distribution and observations are not paired. However, when these criteria are not met, other statistical tests must be used.

## Yates' Continuity Correction

Before proceeding to the other statistical tests for association, it may be interesting to point out a modification to the χ2 test suggested by Frank Yates, an English statistician, in 1934^[4]^. The traditional χ2 test of association assumes a continuous probability distribution to approximate discrete probabilities; this assumption can lead to error. In order to reduce this error, Yates suggested a correction consisting of subtracting 0.5 from the difference between each observed and respective expected value before running the χ2 test. Although the use of the Yates' continuity correction is a theme of discussion, most authors agree that it should always be used for 2X2 contingency tables. For tables with more than two rows and two columns, however, it should not be used. You do not need to make the extra calculations to perform the χ2 test of association with Yates' continuity correction, most statistical software already offer this possibility among the available tests for association.

## Fisher's Exact Test

The Fisher's exact test is a test of association indicated to cases in which the sample is non-parametric i.e. does not follow the normal distribution or if the sample size is small so that the value in each cell is even smaller. The concept of small sample size is complex, subjective and relative, but we suggest you consider to use the Fisher's exact test when the number of subjects is smaller than 100, if the expected frequencies for each cell is smaller than 5 in 20% or more of them, or if the observed frequency in any cell is zero. In fact, it is never wrong to use the Fisher's exact test for unpaired data, even in situations where the χ2 test can be used.

The concept behind the Fisher's exact test is to determine all the possible combinations of values that result in the same marginal totals as the table of observed frequencies and, then, to calculate the probability that the actual observed values were found among all the possibilities. Although it is not necessary to know the formula to perform the Fisher's exact test, considering that you use statistical software, it can be elucidative to understand how the exact probability is achieved. The fact that this test gives the exact probability of the observed values to be found among all the possible combinations is the reason it is called an exact test. Applying the calculation to our example in Table 1, we will also find a two-tailed *P*-value <0.0001 due to the large sample size of the study. For small samples, however, Fisher's exact test tends to exhibit slightly different p-values than those found by the χ2 test of association, but it is always more precise.

## McNemar's Test

Until now, we discussed the use of tests of association in the context of a dependent and independent variables, thus using unpaired data. However, tests of association can also be used to compare two variables found in the same individuals, as for instance when comparing two diagnostic methods. Here, again, let's use an example to make the concept clearer. Greupner et al.^[5]^ compared the use of 64-row computed tomography (CT) with magnetic resonance imaging (MRI) to evaluate left ventricular function. They submitted 36 patients to both exams and observed the frequencies described in Table 3 for wall motion deficit.

**Table 3:** Wall motion deficit as diagnosed by two diagnostic methods.

		MRI		Total
		positive	negative	
CT	positive	18	5	23
	negative	9	4	13
**Total**		27	9	36

n this situation, once that the same patient is being evaluated by two techniques, the data is paired and both the χ2 test of association and the Fisher's exact test do not take pairing in consideration. Then, the appropriate test to be used is the McNemar's test. This test uses the frequencies of the discordant pairs (+/- and -/+) to calculate a χ2 value, which can be compared to the χ2 distribution for one degree of freedom to obtain the *P*-value. The formula used to calculate the χ2 value is very simple, still, it is not necessary to know it if you use a statistical software (what we strongly recommend!) or even, as mentioned previously, a free an online tool^[3]^. n our example, the two-tailed *P*-value is 0.4227, showing that there is no statistically significant difference between the methods used to evaluate left ventricular function. One important observation is that, differently from the χ2 test of association and the Fisher's exact test, McNemar's test can only be performed in 2X2 contingency tables. Another observation is that the sum of discordant pairs in the sample should be at least 10 to allow McNemar's test to be performed.

## Sign Test

The last test of association we will discuss in this editorial is the Sign test. This test is a very simple non-parametric paired test to compare situations in which the data can be expressed as a plus or a minus sign (what justify its name), representing an increase or a decrease of the dependent variable, not taking into consideration the magnitude of this variation. It can be considered as a simplified alternative to comparison tests for numeric variables, which will be discussed in the next editorial. Still, if a variable can be described as a quantitative value, one should always prefer to use a comparison test for numeric variables (*e.g.* paired t-test, Wilcoxon signed-rank test) over the Sign test, which should be reserved for situations in which the quantification of the variable is difficult or not possible.

Again, making use of an example to facilitate the comprehension, suppose you are investigating the effect of an analgesic drug in patients undergoing cardiovascular surgery: you include in your sample 50 patients and, after administering the drug, you find that of those, 30 patients reported improving in pain, 5 patients did not observe any difference, and 15 patients reported worsening in pain. In this case, you can consider you have 30 plus signs and 15 minus signs; the zeros must be discarded in the Sign test and, thus, your sample size is now 45. Having the number of plus and minus signs and the size of the sample, it is possible to calculate the p-value for this association. This calculation includes the use of a standard binomial test to compare the observed data to the binomial distribution. The details for this calculation will not be described here. Most statistical packages offer the possibility to calculate the Sign test, but, again, free online tools are also available to be used^[3]^. In our example, the two-tailed *P*-value is 0.0357, meaning that the administration of the drug is significantly associated with improvement in pain. 

## Assessing Risk

So, now you know which test to choose and how to find statistically significant associations between two qualitative variables. The tests described above, however, can only tell if there is an association, but cannot quantify or point to the direction of it - except for the Sign test. To make that we use measures of risk. Measures of risk represent the probability of occurrence of an event or outcome and it can appear in two forms: risk and odds.

## Risk, Odds, Relative Risk, and Odds Ratio

Risk, itself, is defined as the likelihood to develop an outcome if exposed to a risk factor. Mathematically, it is the ratio of the exposed subjects who present the outcome over all the exposed subjects. Another term often used to refer to risk is odds. Odds is the ratio between the probability of the subject exposed to a risk factor to develop an outcome and the probability of not developing it. It can be calculated by simply dividing the number of exposed subjects who developed the outcome by the number of exposed subjects who did not develop the outcome. Although these two measures of risk may seem similar and, sometimes, are even used interchangeably, there are considerable differences which will impact on the appropriate use of them.

Let's use our first example to illustrate the use of risk and odds. Taking into consideration the patients submitted to CABG with and without AXC and the need for PVS, it is possible to calculate the risk and the odds of needing PVS after each type of procedure (Table 4). It is possible to observe how different risk and odds can be, particularly for frequent events. In fact, the rarest is an event, the most similar is the risk and odds for that event. So, if odds is not always representative of risk, why would one use it? We will discuss that in a moment, but before, we must introduce two measures frequently used to compare risks: the relative risk and the odds ratio.

**Table 4:** Risk and odds.

	PVS	No PVS	n	Risk	Odds
CABG with AXC	489	499	988	489÷988 = 0.49	489÷499 = 0.98
CABG without AXC	43	114	157	43÷157 = 0.27	43÷114 = 0.38

The relative risk (RR) is the ratio between two risks, the risk of the intervention or experimental treatment (the exposed group) over the risk of the control (the group not exposed). The odds ratio (OR), in turn, works exactly in the same ways, but, instead of being the ratio of the risks, is the ratio of the odds. For both the RR and the OR, if the ratio is below 1, it means the risk/odds is lower in the exposed group, if the ratio is greater than 1, the risk/odds is higher in the exposed group. Logically, if the ratio is exactly 1, there is no difference in the chance to develop the outcome between the exposed and not exposed groups. When we work with statistics, however, we can never trust in a single and exact number whether it is the mean, the median, or even a ratio as RR and OR. We should always work with confidence intervals. Thus, what we actually do to affirm if there is or there is not a difference (be it a reduction or an increase) between the risks of two different treatments is to define the confidence interval (CI, usually the 95% confidence interval) of the RR or OR - what can be easily done using a statistical software - and, if the value 1 is included within this interval, we consider there is no difference between the groups. If the value 1 is not in the interval, we can say that the RR or OR of the exposed group is lower (if the CI is below 1) or higher (if the CI is above 1) than the not exposed group.

In Table 5, you can observe that the RR of PVS in the group without AXC (this is the experimental group in the study) versus the group with AXC (the control) is 0.55 i.e. 55% (95%CI: 0.43-0.72). This means that by not using AXC there is a 45% (100% minus 55%) decrease in the risk of developing PVS. The OR, in turn, is 0.39 i.e. 39% (95%CI: 0.27-0.56), meaning CABG without AXC reduces the odds of developing PVS by 61% compared to traditional surgery with AXC. It is interesting to note that the OR, compared to the RR, is a measure that exaggerates the strength of the association between the dependent and independent variables *i.e.* the risk factor and the outcome. When RR is 1 OR is also 1, but the farther the RR is from 1, the farthest is the OR from 1 so that after some degree of increase or decrease in the RR, both measures are too different to be used interchangeably, as it is the case in our example. In fact, the use of OR should be preferably reserved to those outcomes with a frequency in the sample of less than 10%.

**Table 5:** Relative risk and odds ratio.

	PVS	No PVS	n	Risk	Odds	Relative risk (RR)	Odds ratio (OR)
CABG with AXC	489	499	988	0.49	0.98	0.27÷0.49 = 0.55 (95%CI: 0.43-0.72)	0.38÷0.98 = 0.39 (95%CI :0.27-0.56)
CABG without AXC	43	114	157	0.27	0.38		

Now that it is clear what is and how to calculate the RR and the OR, let's go back to that question: If odds is not always representative of risk, why would one use it? The answer is that, for calculating risk, you need to know the total number of subjects exposed to the risk factor, while for calculating odds you just need to know the number of subjects who developed or not the outcome. In case-control studies, the total number of exposed subjects is not available, because you select them based on the occurrence of the outcome and not on the exposure to the risk factor. Differently, in our example, patients were selected based on the exposure to two types of procedure (CABG with and without AXC) and then the frequency of events (PVS) was calculated so that you know the total number of exposed subjects. If we selected a sample of patients who developed PVS as "case" and those who did not develop PVS as "control" among patients undergoing CABG, we would be arbitrarily dictating the number of subjects with and without PVS and therefore the RR, which would not be representative of the RR for the whole population. This is the reason the OR must be used for case-control studies because the RR cannot be calculated for this type of methodological approach.
